# High-speed, automatic dispenser/diluter or dual pipetter/mixer

**DOI:** 10.1155/S1463924678000061

**Published:** 1978

**Authors:** David L. Krottinger, Michael S. McCracken, Howard V. Malmstadt

**Affiliations:** 1 School of Chemical Sciences University of Illinois at Urbana-Champaign Urbana Illinois 61801 USA; 2 Continental Oil Co. Analytical Research Section Ponca City OK 74601 USA; 3 The Proctor & Gamble Co. Miami Valley Laboratory Cincinnati OH 45200 USA


have considered this aspect in some detail for the analysis of vitamins. Accepted manual methods often do not correlate well with automatic analysis primarily because the operating conditions employed in a manual analysis lack the control of the automatic systems. The other significant problem is that components used in commercial instruments or which are readily available are not sufficiently inert to handle corrosive materials often required for an automated procedure. In the automated digestion system described by Jackson et al [16], where concentrated nitric and sulphuric acid are used to destroy the organic matrix of foodstuffs prior to trace metal analysis several components had to be re-designed and developed in-house [17-19]. In addition, a unique displacement pumping arrangement was devised to introduce a slurry of partially digested food quantitatively into the spinning digestor helix. As with the work of Roy [15] with vitamins an in depth appreciation of the reactions involved was also essential to complete the programme of work and to obtain meaningful results.

This paper has centred on the problems of automation in the area of industrial analytical chemistry, however, it is clear that whilst being a multidisciplinary subject, automatic chemistry also transcends disciplinary boundaries. There seems considerable merit therefore in the cross fertilisation of ideas and concepts between the clinical chemists, the process control chemist and the industrial chemist. Such transfer of ideas and evolvement of an acceptable philosophy can only be of benefit to all concerned, and will assist the development of unified philosophies of automatic analysis.

REFERENCES

[1] Skeggs, L. T., *American Journal of Clinical Pathology,* 1957, **28,** 311.
[2] Anderson, M. G., *American Journal of Clinical Pathology,* 1970, **53,** 778.
[3] Ruzicka, J. and Hansen, E. H., *Analytica Chimica Acta,* 1957, **78,** 31.
[4] Deans, D. R., *Proceedings of The Analytical Division of The Chemical Society,* 1977, **14,** 199.
[5] Arndt, R. W., Werder, R. and Fres, Z., *Analytical Chemistry,* 1977, **278,** 15.
[6] Ruzicka, J., Hansen, E. H. and Reetz, B., *Analytica Chimica Acta,* 1977, **89,** 24.
[7] Stewart, K. K., Beecher, G. R. and Hare, P. E., *Analytical Biochemist,* 1976, **79,** 162.
[8] Karlberg, B. and Thelander, S., *Analytica Chimica Acta,* 1978, **89,** 1.
[9] Stockwell, P. B. and Sawyer, R., *Analytical Chemistry,* 1970, **42,** 1136.
[10] Burns, D. A., Seventh Technicon International Congress, New York 1976, *Proceedings* 1977.
[11] Morley, F. and Stockwell, P. B., *Journal of Dentistry,* 1977, **5,** 39.
[12] Lidzey, R. G. and Stockwell, P. B., *The Analyst,* 1974, **99,** 749.
[13] Norris, K. H., and Hart, J. R., *Proceedings 1963 International Symposium on Humidity and Moisture,* Peinhold, New York, 1965, **4,** 19.
[14] Renoe, B. W., O'Keefe, K. R. and Malmstadt, H. V., *Analytical Chemistry,* 1976, **48,** 661.
[15] Roy, R. B., Conetta, A. and Salpeter, J., *Journal of The Association of Official Analytical Chemists,* 1976, **59,** 1244.
[16] Jackson, C. J., Porter, D. G., Dennis, A. L. and Stockwell, P. B., *The Analyst,* 1978, **103,** 317.
[17] Porter, D. G., Jackson, C. J. and Bunting, W., *Laboratory Practice,* 1974, **23,** 111.
[18] Lidzey, R. G., Jackson, C. J. and Porter, D. J., *Laboratory Practice,* 1977, **26,** 400.
[19] Jackson, C. J., Morley, F. and Porter, D. G., *Laboratory Practice,* 1975, **24,** 23.

### High-speed, automatic dispenser/diluter or dual pipetter/mixer

**David L. Krottinger***
**Michael S. McCracken****
**Howard V. Malmstadt†**

*School of Chemical Sciences, University of Illinois at Urbana-Champaign, Urbana, Illinois 61801, USA.*

**ON** several of our automated analytical systems [1, 2, 3] we have need for a device that can either operate as a precise dispenser/diluter of discrete quantities of solution or can be used to pipet automatically both a sample solution and a reagent and efficiently mix them as the sample-reagent solution is rapidly and efficiently mix them as the sample-reagent solution is rapidly delivered into a measurement cell. Although there are commercial systems on the market, none have the combination of high-speed refill/delivery/mixing, modest cost, and automated features that we require. Consequently, we have developed a portable stand-alone unit that refills, delivers and mixes solutions in ways quite similar to some stopped-flow instruments. Experience with this type of automated unit for more than one year has provided ample data to demonstrate its general utility and reliability for the analytical laboratory. Therefore, we present here the details of the unit, which constructed primarily from commercially-available parts, and also some test results to indicate its performance characteristics.

*Present Address: Continental Oil Co., Analytical Research Section, Ponca City, OK 74601.
**Present Address: The Proctor & Gamble Co., Miami Valley Laboratory, Cincinnati, OH 45200.
†Address correspondence to this author.

### Instrumentation

The design of the dual pipetter/mixer or dispenser/diluter is based on the principles illustrated in Figure 1. Pneumatically-operated syringes together with pneumatically-actuated, 3-way KEL-F valves enable the syringes to be filled and the solutions to be delivered by electronic control signals, and an X-Y mixer in the delivery channel to the measurement cell provides the mixing of the two solutions.

The completely assembled unit is shown in Figure 2, and the partially-assembled unit showing the framework for mounting the syringes and valves and drive system is shown in Figure 3. An exploded view of the unit illustrating the interconnections of each of the components is shown in Figure 4 with the components that were used in the construction listed in Table 1. More detailed description follows.

### Dual, 3-way valves

Two commercially available, 3-way valves were used in the unit to provide for automatic control of the solution flow. Both 3-way valves contain an inlet connection to the solution reservoir, a syringe port in which the desired syringe assembly is mounted, and an outlet port for the solution to be delivered from the syringes. A KEL-F slider is pneumatically positioned in each valve to interconnect the desired ports. These valves have an internal volume of only 42$\mu$l providing for low dead volumes. This allows the use of the unit for applications where limited volumes of solutions are available for applications requiring expensive reagents. The small dead volume also enables rapid changeover of reagents or diluents. For applications not requiring the low dead volume, solenoid operated valves can be used thereby eliminating the need for the 75 psi required for the pneumatic valves. A KEL-F valve body was chosen for our application because of the inert nature of KEL-F to the strongly acidic solutions that were present in some of our applications. A nylon body can be used for applications where the inertness of KEL-F is not necessary and the added expense not warranted. The bodies of the valves were used as provided by the manufacturer with the sliders modified for our needs. It was desired for our applications that the solutions be aliquoted through the top of the valves and into the syringes and that the delivery step take place by passing the solution straight through the valve into the X-Y mixer. To facilitate this, the delivery position of the commercial KEL-F sliders must be modified as shown in Figure 5A. This was achieved by plugging the vertical channel in the delivery position and extending the horizontal channel completely through the slider. The fill position of the sliders was used as provided by the manufacturer.

The dual 3-way valves are controlled by positioning the slider with a pneumatic actuator and a spring return. In the deactivated state of the pneumatic actuator, the spring return positions the sliders such that the syringes are connected to the solution reservoirs. The syringes are connected with the X-Y mixer in Figure 4 by application of 75 psi to the pneumatic actuator, thereby compressing the spring and moving the slider to the deliver position. A subminiature solenoid valve (Angar Scientific Corp., East Hanover, New Jersey 07936) controls the supply of compressed air. A small screw is added to the spring return to encode the position of the slider. When the sliders are in the deliver position, the screw mounted on the valve interrupts the light emitting diode signal to the phototransistor in the optical interrupter as shown in Figure 6. This technique allows for feedback in the control circuitry to ensure that delivery of the aliquoted solutions does not begin until the pneumatic actuator has moved the sliders into the delivery position.

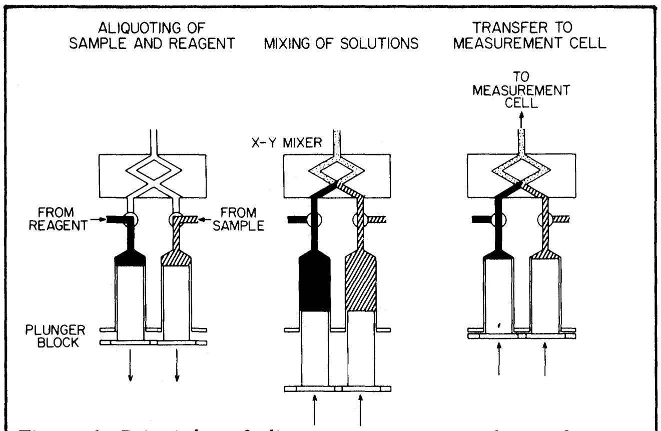

*Figure 1. Principles of aliquoting, mixing, and transfer operations*

To provide the 75 psi needed to compresss the spring return mechanism a convenient, portable source of compressed air was constructed with a commercially available compressor (Coleman Inflate-all 90, Model 2239A, Coleman Company, Inc., Wichita, Kansas 67201) and a 5 ½ litre ballast tank. The air compressor was used to fill the ballast tank with sufficient air for 100 cycles of the module. Alternatively a small commercial compressed air tank can be used.

**Syringes and holders**

A variety of commercial syringes were evaluated to determine which would require the least maintenance. The most viable candidates were found to be the Glenco gas/liquid syringe series. The plunger tip consists of a TFE piece under constant tension from an internal silicone rubber core to reduce the frequency of leaks.

The syringe assembly is constructed as shown in Figures 4 and 5B to provide a secure mount. The Glenco syringe barrels are modified by cutting and polishing the ends as shown in Figure 5B. Care must be taken to ensure that the cut is perpendicular to the syringe barrel. The Unimetrics TFE inserts are pressed into the front end of the syringe barrel to provide a

*Figure 2. Completely assembled unit*

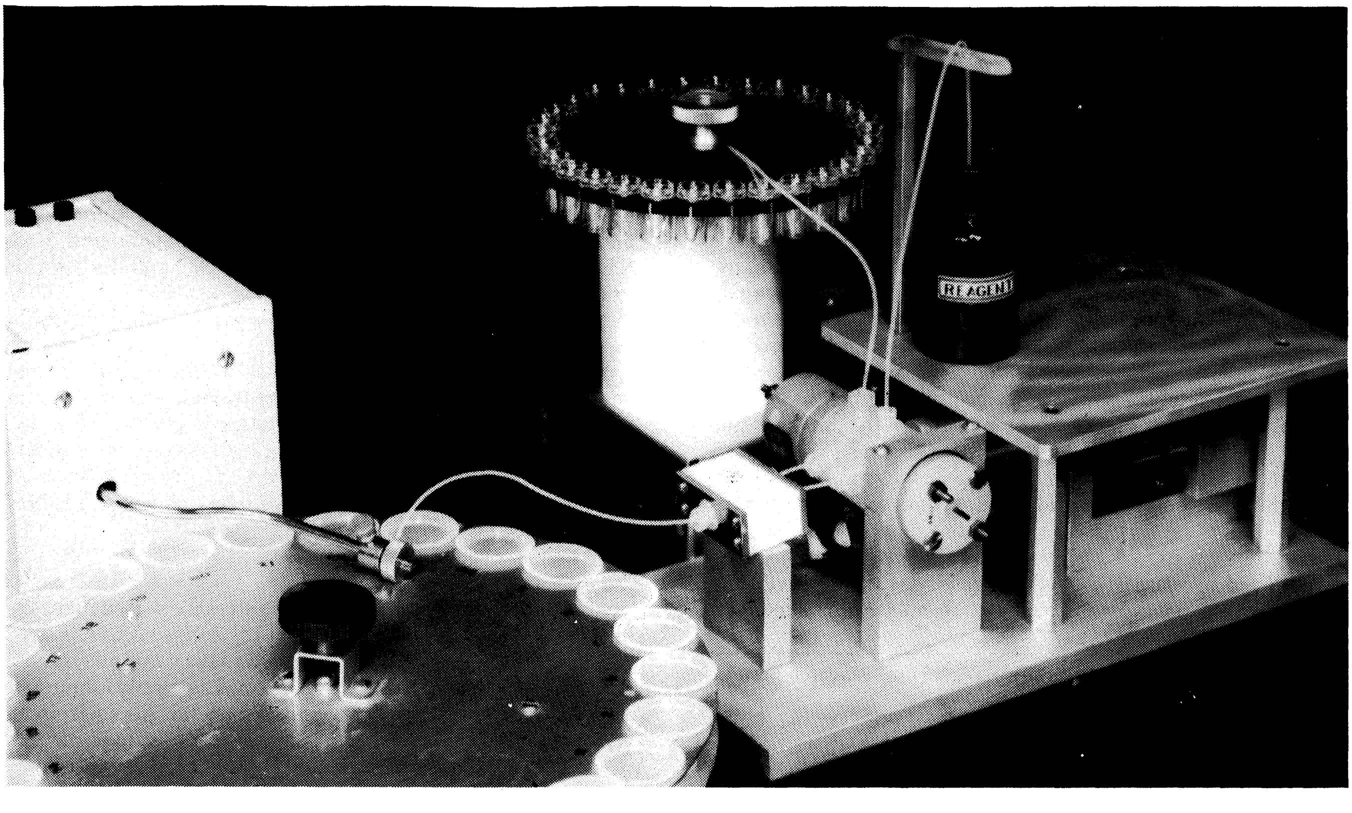

leak-free seal. This syringe barrel is then placed into the aluminum holder.

The holder is made from a ⅝″ diameter aluminum rod which has been bored to within ¼″ of the end of the rod to accept the 11/32″ diameter syringe barrel. After a syringe barrel has been inserted, the syringe stop is screwed into the back end of the holder, which is tapped to accept ⅜″-24 threads. A one-inch piece of ¼″-28 threaded KEL-F rod is screwed into the front end of the holder, tightly against the TFE insert in the syringe barrel to eliminate leakage. Each syringe assembly is screwed into the valve and the back end of the plungers screwed into the plunger block.

### Syringe drive system

The two syringe assemblies are driven by a double-acting air cylinder (Mosier Industries, Inc.) with a ¾ inch bore and a 2 ½ inch stroke. The cylinder is controlled by two subminiature solenoid valves as used with the pneumatic actuator. The air cylinder movement is coupled to the syringe plungers by the plunger block which provides for simultaneous movement of both syringes during the filling and dispensing processes. The length of the stroke of the air cylinder may be varied by the use of a mechanical stop to adjust the volume delivered by the unit for various applications.

The pneumatic type of driving system was chosen because of its flexibility and size compared to spring loaded or motor driven mechanisms. Miniature air regulators and gauges (Walter Norris Engineering Co., Chicago, IL 60648) are used to provide 18 psi for filling of the syringes and 24 psi for delivering the solution aliquots. The 18 psi fill pressure allows for a one second aliquot step. Higher fill pressures decrease the time required to fill the syringes, but also result in degassing of the solutions which is undesirable for high-accuracy applications. The 24 psi provides for adequate delivery pressure for efficient mixing of the solutions as they pass through the X-Y mixer. A schematic of the compressed air system is shown in Figure 7. The 75 psi needed for the pneumatic actuator is used with the

**Table 1 Component list for dual pipetter/mixer and dispenser/diluter**

| *Component* | *Model No.* | *Supplier* |
|---|---|---|
| 250 μl syringe | 19925-025 | Glenco Scientific Co. Houston, TX 77007 |
| 500 μl syringe | 19925-05 | Glenco Scientific Co. |
| 1000 μl syringe | 19925-1 | Glenco Scientific Co. |
| Syringe plungers | 19925-P series | Glenco Scientific Co. |
| Teflon insert for syringes | Unimetrics Corp. 4000 needle seals | Utopia Instrument Co. Joliet, IL 60434 |
| Pneumatic actuator | PA-875K | Lab Data Control Riviera Beach, FL 33404 |
| 2 - 3-way Slider valves | CAV3031K | Lab Data Control |
| spring return | SR1K | Lab Data Control |
| Tube end fittings for ⅛″ O.D. Teflon tubing | TEF-107 | Lab Data Control |
| ⅛″ O.D. teflon (0.8 M.I.D.) | TO63031 | Lab Data Control |
| Air cylinder | Mosier Industries Model TSR ¾″ bore x 2 ½″ stroke | J. N. Fauver Co. Schaumburg, IL 60172 |
| Miniature regulators | C. A. Norgen Co. RO4-100-RNK-AU | Walter Norris Eng. Co. Chicago, IL 60648 |
| 0-100 PSI gauges | C. A. Norgen Co. 18-013-237 | Walter Norris Eng. Co. |
| Subminiature solenoid valves | 3N06B6 | Angar Scientific Corp. East Hanover, NJ 07936 |
| Ball bushings | Thomson Ind. Inc. Super 4 | Danville Bearing & Supply, Danville, IL 61832 |
| Bushing shafts | Thomson Ind. Inc. 60 Case | Danville Bearing & Supply |
| Oil-impregnated bronze bushing | | Boston Gear Co. Quincy, MA 02171 |
| Air compressor | Inflate All 90 2239A | Coleman Co., Inc. Wichita, Kansas 67201 |

*Figure 3. Syringe, valve, and drive system of the unit*

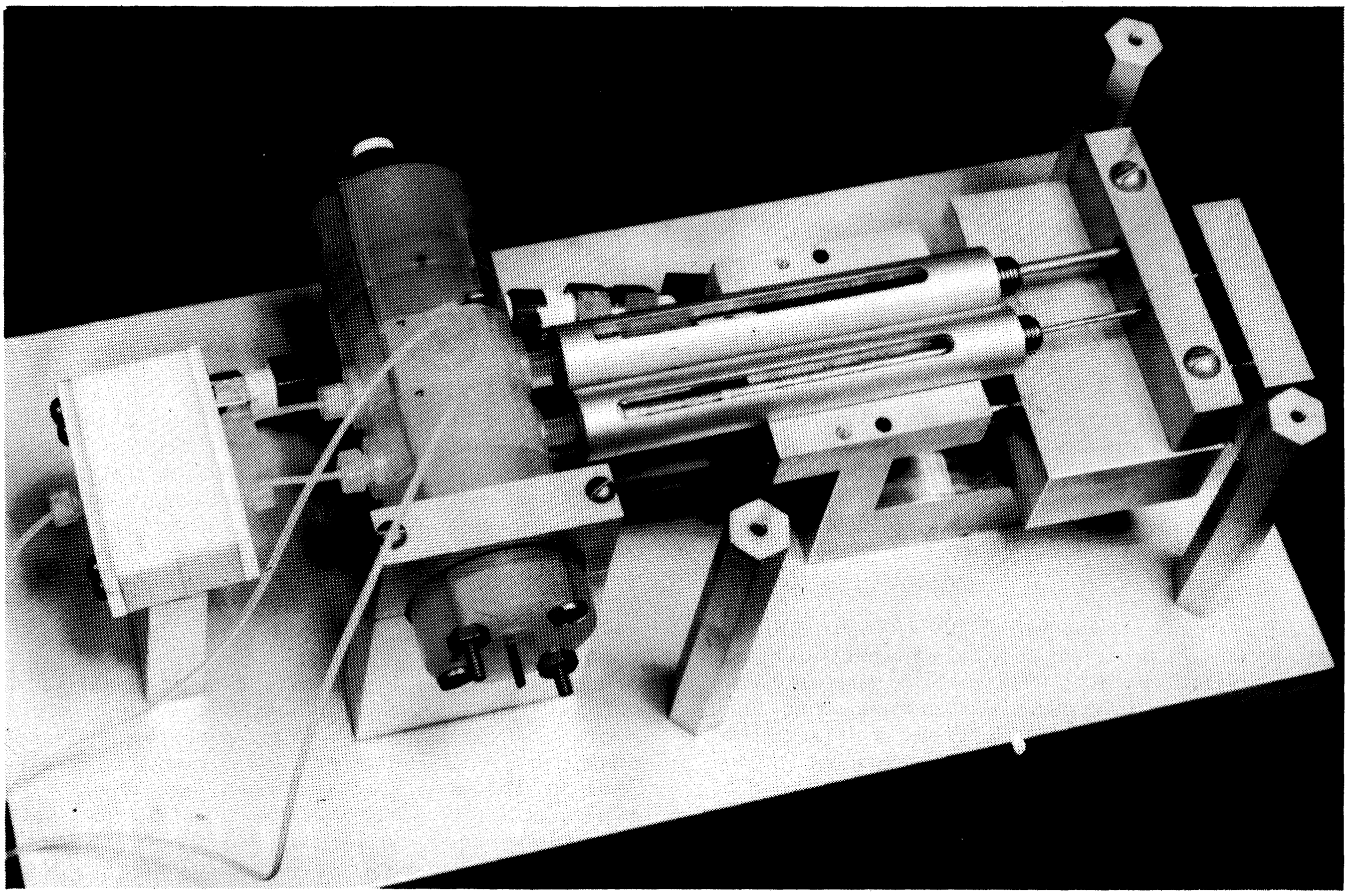

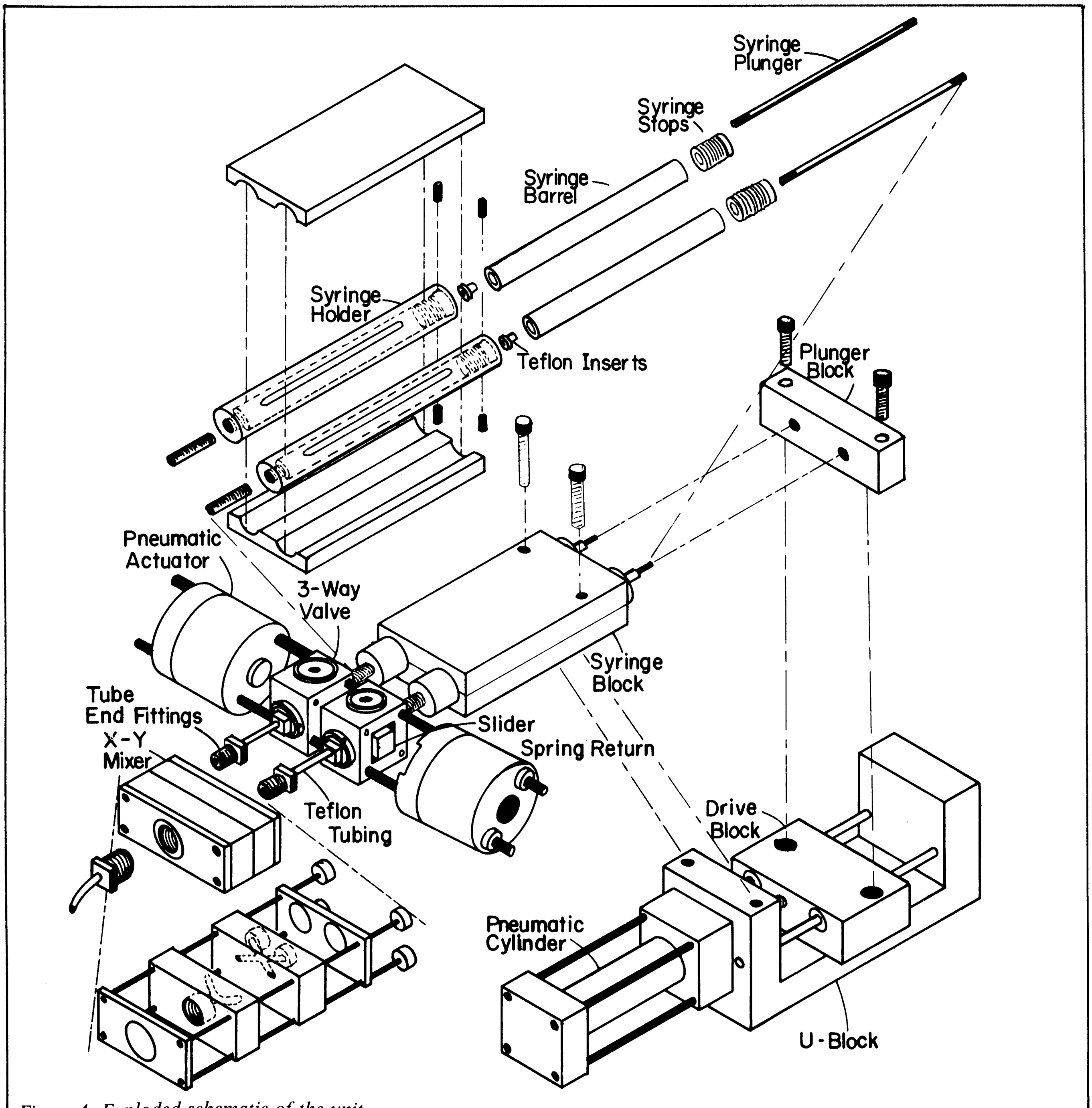

*Figure 4. Exploded schematic of the unit*

miniature regulators to provide the compressed air needed for the fill and deliver ports of the air cylinder. Both ports of the air cylinder and the pneumatic actuator are activated by separate subminiature solenoid valves at the correct time by the control circuitry to provide for an automatic sequencing of the fill and deliver steps of the unit.

### Mixer

The X-Y mixing chamber shown in Figure 4 is constructed from two pieces of TFE containing an X and a Y channel for mixing of the solutions. The channel diameters in the mixer are 0.8 mm to be compatable with the valve and the interconnecting tubing used in the unit. TFE was chosen because of its inertness to caustic solutions used in some of our applications and the good seal that is formed between the interface of the two halves of the mixer when clamped together with two aluminum plates. The ports connecting the mixer and valve and the receiving vessel or measurement cuvet are constructed to accept the commercial ¼″-28 tube end fittings which are used throughout the unit.

The X-Y configuration was chosen based on studies conducted in our laboratory that indicate that for our applications, the X-Y mixing configuration provides good mixing of solutions and a lower dead volume than found in more involved mixing arrangements [4]. The dead volume of the mixer depends on the length of the X and Y channels which for this case resulted in a dead volume of 53 $\mu$l.

### Control circuitry

A schematic diagram of the control circuitry for the unit is shown in Figure 8. Normal operations of the module begin with placement of the tubing from the valve into the appropriate solution reservoirs or sample cup. The syringes are filled from the solution reservoirs by applying a logic level "O" to monostable 1 (M1) which results in solenoid 1 (S1) being activated for one second and the plunger block moving the plungers back. To deliver the solutions from the syringes, the 3-

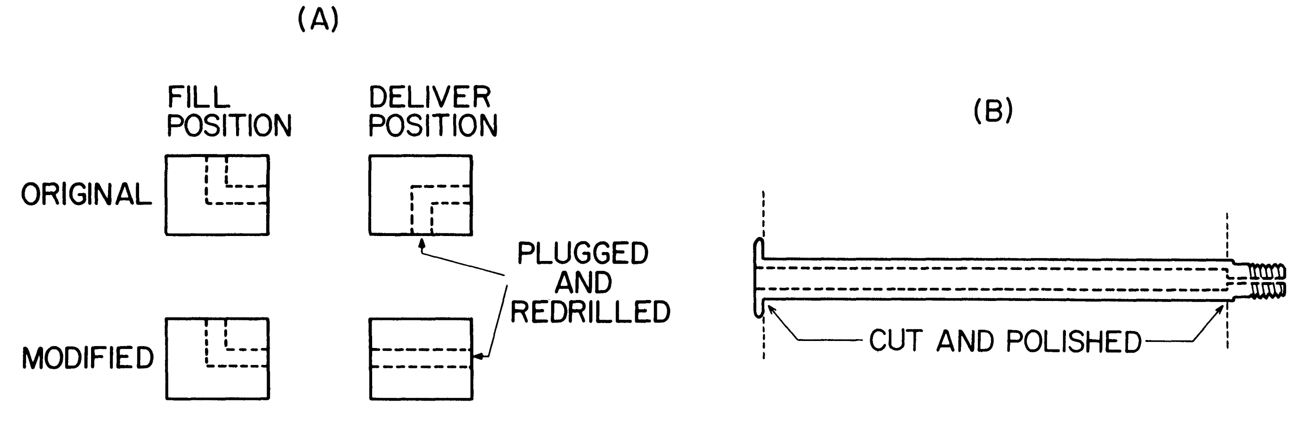

*Figure 5. Modifications to commercial components (A) flow passage through sliders; (B) syringe barrel*

**Table 2 Gravimetric data for the pipetter/diluter**

| *Syringe capacity (μl)* | *Volume delivered (mg)* | *%RSD** |
|---|---|---|
| 250 | 150.14 | 0.05 |
| 500 | 288.41 | 0.03 |
| 1000 | 565.71 | 0.08 |

*Based on 10 individual cycles

**Table 3 Spectrophotometric data for the pipetter/diluter**

| *Dilution ratio* | *Absorbance readings*† | *%RSD** |
|---|---|---|
| 1:1 | 0.553 | 0.11 |
| 1:2 | 0.614 | 0.10 |
| 2:1 | 0.618 | 0.11 |
| 1:4 | 0.562 | 0.12 |
| 4:1 | 0.603 | 0.11 |
| no cycles | 0.552 | 0.10 |

*Based on 10 individual absorbance measurements

†Blue dextran solution prepared for each dilution ratio so that the absorbance after dilution was approximately 0.6 in the 1-cm cell at 620 nm.

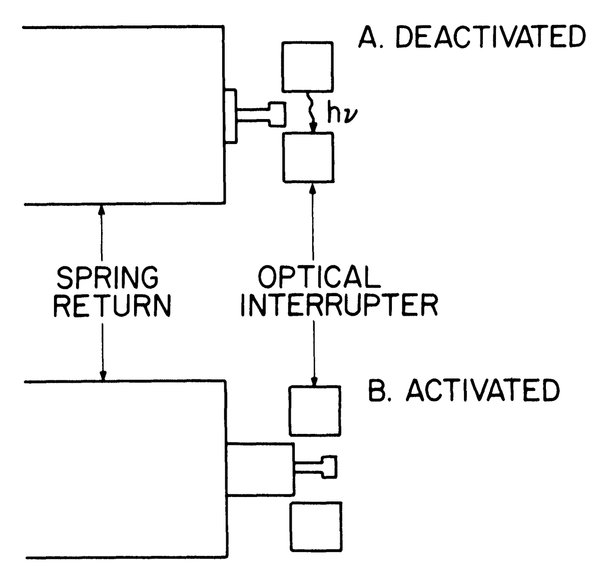

*Figure 6. Encoding of valve position*

way valve must first be actuated and when this is completed, the plungers are then driven forward. When the flip-flop (FF) receives a logic level "O", solenoid 2 (S2) is turned on resulting in the valve being actuated. An optical interrupter assembly (O.I.) mounted on the end of the spring return of the valve detects when the sliders in the valve have moved to the delivery position. When the sliders contained in the valve move to the delivery position, a screw mounted on the valve interrupts the light emitting diode signal to the phototransistor in the optical interrupter. This status of the valve is digitally encoded by the Schmitt Trigger (S.T.) resulting in monostable 2 (M2) turning on solenoid 3 (S3) for one second. This results in the delivery of the solution. At the end of the one second delivery, the flip-flop is cleared and the valve returns to connect the syringes to the original solution reservoirs. The unit can function in a manual mode in which an operator causes the aliquoting and delivery of solutions or it can function as part of an automated system in which the control of the pipetter/diluter is performed by a minicomputer or microprocessor.

### System evaluation

### Reagents

A stock solution of blue dextran 2000 (Pharmcia Fine Chemicals, Inc., Piscataway, NJ 08854) was prepared by dissolving 1 gram of the solid in 100 ml of distilled water.

### Dispenser/diluter characteristics

The process of obtaining an aliquot of a sample, mixing it with a suitable diluent, and transferring the resulting mixture to a suitable vessel is a procedure used in most analytical labs. This step can be a major source of error if performed manually due to the multiple times that the sample must be manipulated. The unit described in this paper can perform this type of procedure rapidly and precisely providing a valuable tool for analytical chemists. To demonstrate the reliability of the unit a number of tests were run. The first evaluation of the unit as a dispenser/diluter was done to determine the precision with which solution could be aliquoted and delivered from various syringes. To do this, aliquots of water were repetitively delivered into a weighing bottle on an analytical balance with readings taken after the addition of each aliquot of solution. Results obtained for the unit in this dispenser mode for various syringes are shown in Table 2. The data illustrate the excellent precision with which the unit can deliver from 250 μl to 1 ml of solution.

The ability of the unit to operate as a dispenser/diluter was determined by mixing a blue dextran solution with water and measuring the resulting absorbance in a ratio-recording spectrophotometer (Model 721, GCA/McPherson, Acton, MASS 01720). This was accomplished by use of a 1-cm flow cell (Model T-72, Precision Cells, Inc., Hicksville, NY 11801) connected to the outlet of the mixer. The blue dextran solutions used for each dilution ratio were prepared so that the absorbance after dilution by the unit was approximately 0.6 when measured in the one centimeter cell at 620 nm. Five absorbance readings with a one-second measurement time for each were averaged to form the absorbance reading for one cycle of the unit. A ten-second delay after delivery of the solutions was employed to allow for any turbulence of mixing

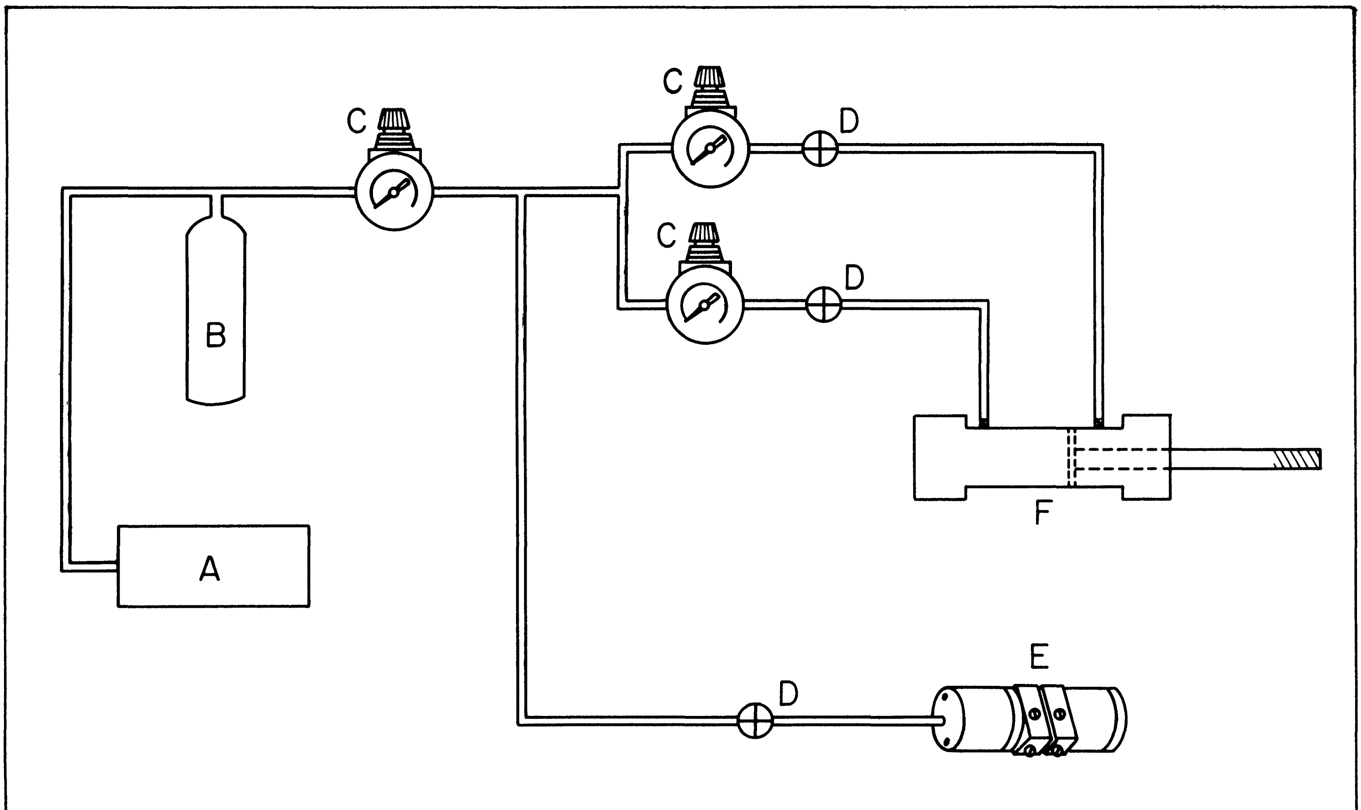

*Figure 7. Schematic of compressed air system. (A) air compressor; (B) ballast tank; (C) miniature regulators; (D) solenoid valves; (E) pneumatic actuated valves; (F) air cylinders*

to subside prior to taking the absorbance measurement for a given cycle.

The unit was evaluated with a number of different dilution ratios as shown in Table 3. As indicated in the data, this unit serves as a precise means of aliquoting samples and mixing them with a suitable diluent. The precision of the absorbance readings was equivalent to those obtained with no cycling of the unit between ten successive sets of five absorbance readings indicating that the spectrophotometric measurement was the limiting factor in the obtainable precision.

**Duel pipetter/mixer**

In many analytical techniques it is necessary to obtain aliquots of a sample and a suitable reagent, mix the two, and transfer the mixed solutions to an observation cell or to a receiving vessel for subsequent sample-handling procedures. For example, in some procedures, reagents used for an analytical procedure are unstable as a composite reagent and must be added sequentially to the sample to perform the determination. The unit shown in Figure 2 has a number of desirable features which we have found make it useful for applications that require a dual pipetter/mixer. The unit is versatile in that the syringe assembly described earlier allows for rapid changing of the syringes enabling the use of a wide range of volumes depending on the particular application. The unit also performs the aliquoting, mixing, and transfer operations of the sample and reagent in about two seconds. This enables the rapid changeover from one sample to another. After one sample has been mixed with the reagent and transferred to a suitable vessel, the unit must be purged of the present sample and the next sample prepared. This is done by flushing the next sample through the unit until the previous sample has been removed. The number of cycles and the time required to perform this changeover of samples is dependent on 1) the syringe volume, 2) the length of tubing between the valve and the solution reservoir, 3) the length of tubing between the valve and the mixer, and 4) the length of tubing between the mixer and the measurement cuvet. Changeover data for one of our applications in which a 1000 $\mu$l syringe and a 250 $\mu$l syringe were used to deliver the solution volumes are shown in Figure 9. In this application the sample

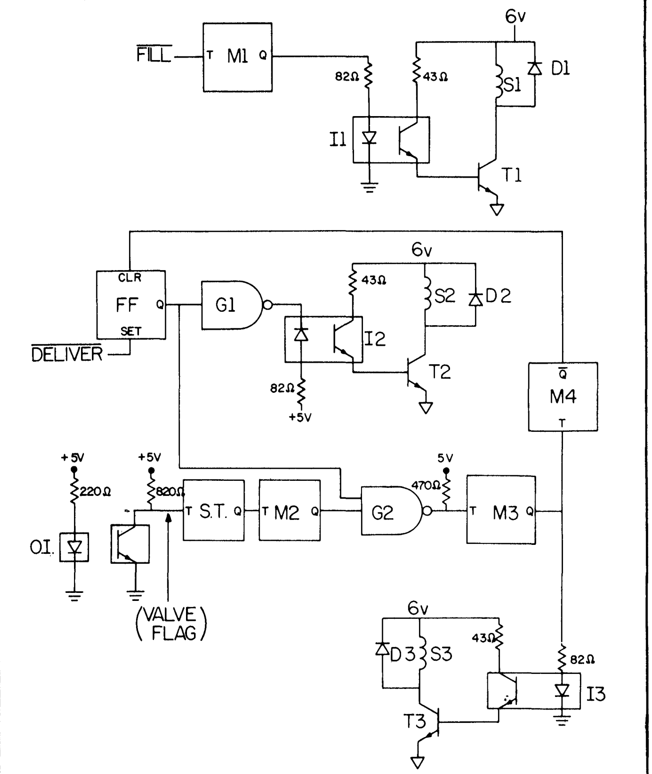

*Figure 8. Schematic of the control circuitry (M1, M3, S.T.: 555; I1-I3: MCT2; T1-T3: 2N2270; D1-D3: IN4148; S1-S3; #3NO6B6, Angar Scientific Corp., East Hanover, NJ 07936; O.I.: GEH13B2; M2, M4: 74123; FF: 7474; G1, G2: 7438; All Resistors are 1/4 Watt, 10%)*

and reagent are aliquoted, mixed and transferred to the flow cell in the spectrophotometer described in the discussion of the dispenser/diluter mode of the unit. To demonstrate the changeover characteristic of the unit, the "sample" present in the unit at cycle O is water as is the "reagent". The cycles after O result from a blue dextran solution serving as the "sample"

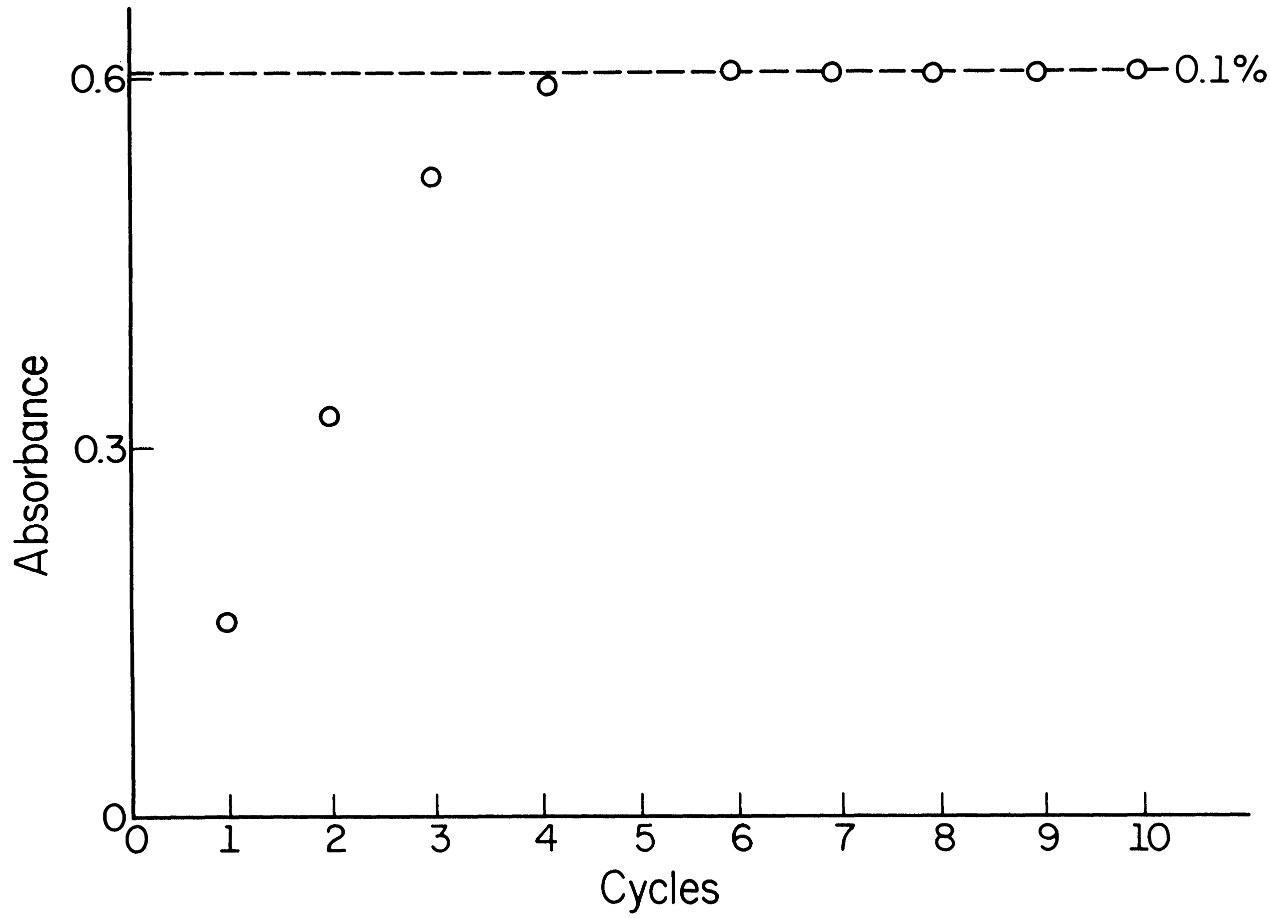

*Figure 9. Changeover data for dual pipetter/mixer*

being aliquoted by the 250$\mu$l syringe. The absorbance readings for each fill and deliver cycle of the unit are obtained at 620 nm as described earlier. As noted in Figure 9, the unit requires for this configuration only 5 cycles and thus 10 seconds to completely change from one sample to another using these syringes. This number of cycles can be further decreased in applications in which the unit is to operate as a dedicated instrument by decreasing the length of the tubing connecting the different components of the unit or increasing the sample syringe volume.

The dual pipetter/mixer has been used for several months in our laboratory in a phosphorus determination for grain and feed samples which have undergone a Kjeldahl digestion. The automated spectrophotometric system [1] used for this determination allows the addition of only one reagent solution to the sample. The reaction-rate methodology used in the phosphorus determination requires the addition of two reagents, a molybdenum solution and an ascorbic acid solution, to form the phosphomolybdenum product which is monitored by the analyzer. As a result of the unstable nature of a single molybdenum-ascorbic acid reagent, the ascorbic acid reagent is first aliquoted and mixed with the sample by the pipetter/mixer and the resulting prepared sample transferred to a programmable turntable of the automated spectrophotometric system at which point the molybdenum reagent is added to the sample-ascorbic acid mixture. Determinations performed by the automated system on 10 samples prepared by the dual pipetter/mixer from a single digestion demonstrate a precision of 0.3% RSD. This same precision is obtained for repetitive determinations by the automated system on one prepared sample thus indicating that the spectrophotometric determination is again the limiting factor in the obtainable precision.

This type of portable pipetter/mixer and dispenser/diluter has provided excellent precision for several applications in our lab over the past year. It has been automated to provide reliable operation as a module in our automated systems and is versatile in the type of applications it can be used for. It also has been used for manual push-button pipetting, diluting and dispensing.

REFERENCES

[1] D. L. Krottinger, M. S. McCracken and H. V. Malmstadt, *American Laboratory*, **9** (3), 51 (1977).

[2] R. W. Spillman and H. V. Malmstadt, *American Laboratory* **8** (3), 89 (1976).

[3] B. W. Renoe, R. P. Gregory IV, J. Avery, and H. V. Malmstadt, *Clinical Chemistry* **20**, 955 (1974).

[4] D. L. Krottinger, M. S. McCracken, and H. V. Malmstadt, *Talanta*, submitted for publication.

